# Pathogenesis of Myeloproliferative Neoplasms: Role and Mechanisms of Chronic Inflammation

**DOI:** 10.1155/2015/145293

**Published:** 2015-10-11

**Authors:** Sylvie Hermouet, Edith Bigot-Corbel, Betty Gardie

**Affiliations:** ^1^Inserm UMR 892, CNRS UMR 6299, Centre de Recherche en Cancérologie Nantes-Angers, Institut de Recherche en Santé, Université de Nantes, 44007 Nantes, France; ^2^Laboratoire d'Hématologie, Centre Hospitalier Universitaire de Nantes, 44093 Nantes Cedex, France; ^3^Laboratoire de Biochimie, Centre Hospitalier Universitaire de Nantes, 44093 Nantes Cedex, France; ^4^Ecole Pratique des Hautes Etudes, Laboratoire de Génétique Oncologique, 44007 Nantes, France

## Abstract

Myeloproliferative neoplasms (MPNs) are a heterogeneous group of clonal diseases characterized by the excessive and chronic production of mature cells from one or several of the myeloid lineages. Recent advances in the biology of MPNs have greatly facilitated their molecular diagnosis since most patients present with mutation(s) in the *JAK2, MPL,* or *CALR* genes. Yet the roles played by these mutations in the pathogenesis and main complications of the different subtypes of MPNs are not fully elucidated. Importantly, chronic inflammation has long been associated with MPN disease and some of the symptoms and complications can be linked to inflammation. Moreover, the JAK inhibitor clinical trials showed that the reduction of symptoms linked to inflammation was beneficial to patients even in the absence of significant decrease in the *JAK2*-V617F mutant load. These observations suggested that part of the inflammation observed in patients with *JAK2*-mutated MPNs may not be the consequence of *JAK2* mutation. The aim of this paper is to review the different aspects of inflammation in MPNs, the molecular mechanisms involved, the role of specific genetic defects, and the evidence that increased production of certain cytokines depends or not on MPN-associated mutations, and to discuss possible nongenetic causes of inflammation.

## 1. Introduction

Chronic myeloproliferative neoplasms (MPNs) are rare hematologic diseases characterized by the clonal proliferation of mature blood elements from several myeloid lineages, associated in certain cases with bone marrow fibrosis, splenomegaly, and/or hepatomegaly. They include chronic myelogenous leukemia (CML), three related entities named polycythemia vera (PV), essential thrombocythemia (ET), and primary myelofibrosis (PMF) (called Philadelphia chromosome-negative (Phi-negative) MPNs), chronic eosinophilic leukaemia, mastocytosis, and unclassifiable MPNs [1]. CML and other MPNs are classified based on the presence or the absence of the* BCR-ABL* fusion gene which is the hallmark of CML [2]. This review focuses solely on Phi-negative MPNs. Three types of molecular markers are associated with Phi-negative MPNs: activating mutations in the* JAK2* gene (*JAK2*-V617F being the most frequent mutation, present in all subtypes of MPNs); activating mutations in the* MPL* gene (*MPL*-W515L/K mostly); and alterations of* CALR*, the gene coding calreticulin (*CALR*), detected in ET and in PMF [3–11]. A small percentage of MPN patients (<15%) do not carry mutations in the* JAK2*,* MPL*, or* CALR* genes.

The exact roles played by* JAK2*,* MPL*, and* CALR* mutations in the pathogenesis, phenotype, and complications of the three MPN subtypes are not fully elucidated. None of the* JAK2*-V617F,* MPL*-W515L/K, or* CALR* mutations is specific of a particular MPN subtype. They are detected in patients with very different phenotype and disease evolution, and therefore their presence alone is not sufficient to explain the clinical presentation and complications observed in MPN patients. Moreover, for subsets of patients, the* JAK2*-V617F mutation has been shown to be a rather late event, sometimes recurrent, which indicates that other genetic events are responsible for clonality in these patients [14–18]. Interestingly, some of the clinical symptoms and complications appear to be linked to the chronic inflammation which almost always accompanies MPN disease, and reduction of symptoms linked to inflammation is beneficial to patients [19, 20]. Presently it is unclear whether the inflammation-related biological markers and clinical symptoms observed in MPN patients are consecutive or reactive to, or perhaps even precede, the main mutations harbored by MPN clones. Obviously, a better understanding of the mechanisms that underlie inflammation in the different MPN subtypes should have a significant impact on the design of future protocols tested for the therapy of MPNs. To help address this issue, the present review describes the role played by somatic as well as germline genetic defects in the increased production of inflammatory cytokines and other inflammation markers in MPNs; potential nongenetic causes of chronic inflammation are also discussed.

## 2. Chronic Inflammation, including Inflammation Associated with Solid Cancer or MPNs

Inflammation is a pathological process typically triggered by an external aggression, which may be a physical or chemical injury, irradiation, or infection. In addition, chronic hypoxia (e.g., when cells accumulate in a solid tumor or in the bone marrow in the context of blood malignancy or in any type of tissue in case of venous or arterial thrombosis) can also lead to inflammation [21–23]. Chronic inflammation is characterized by the prolonged stimulation of the production of immune blood cells from the lymphoid and myeloid lineages and the release of various mediators, notably inflammatory cytokines, in blood vessels and in tissues. Myelopoiesis is stimulated during inflammation so as to produce sufficient quantities of polyclonal granulocytes, monocytes, and macrophages to ensure the destruction of damaged cells, tissues, or infectious pathogens, adequate phagocytosis, and presentation of antigens to lymphocytes. The production of polyclonal megakaryocytes and platelets is frequently increased, to ensure thrombus formation and hemostasis in case of damaged blood vessels in inflamed tissues. Chronic inflammation may lead to hypoxia of variable severity in the damaged tissues and, accordingly, to increased production of polyclonal erythroid progenitors and red blood cells in an effort to improve cell and tissue oxygenation. Conversely, hypoxia can lead to increased production of inflammatory cytokines: individuals with mountain sickness present with elevated levels of inflammatory cytokines in peripheral blood, and healthy volunteers exposed to a hypoxic environment (three nights in high altitude above 3400 meters) presented with a high level of interleukin- (IL-) 6 [24, 25]. Patients with Chuvash polycythemia associated with homozygous germline mutation in the Von Hippel-Lindau (*VHL*) gene, a major actor of the hypoxia sensing pathway, present with elevated levels of tumor necrosis factor- (TNF-) *α* and interferon- (IFN-) *γ* [26]. Inflammatory diseases such as inflammatory bowel disease and rheumatoid arthritis also provide evidence of cross talk between hypoxia and inflammation [27]. In rheumatoid arthritis, hypoxia-inducible factor- (HIF-) 2*α* is the HIF isoform that plays a major role in inflammation, notably by inducing expression of IL-6 and TNF-*α* [28]. Importantly, HIF-1*α* plays an essential role in survival and function of myeloid cells during inflammation [29].

**Figure 1:**
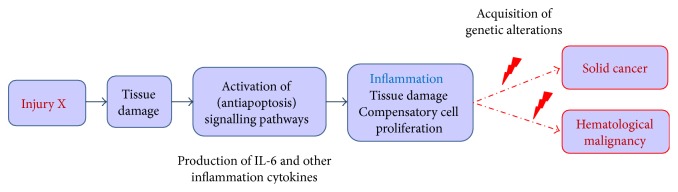
Progression from chronic inflammation to solid and blood cancers. A physical, chemical, or infectious injury leads to tissue and cell damage and activation of antiapoptosis signaling pathways in affected cells, which results in the autocrine and paracrine production and consumption of prosurvival, inflammatory cytokines, as well as chemokines, to attract immune cells of the lymphoid and myeloid lineages to the site of injury. Over time, established inflammation (chronic inflammation) constantly overstimulates the production of hematopoietic cells and induces more tissue and cell damage, hereby increasing the rate of DNA duplication and risk of defective DNA reparation and mutation, both in cells from affected tissues (increased risk of solid cancer) and in lymphoid and myeloid cells participating in the immune/inflammatory response (increased risk of hematological malignancy).

If the initial “injury” persists, the inflammation response and associated chronic stimulation of hematopoiesis are prolonged, and the risk of DNA alteration increases in cells from the damaged tissues or/and in overstimulated hematopoietic progenitors. Over time the acquisition of genetic defects in the inflamed tissues or/and hematopoietic progenitors may eventually lead to the development of solid cancer or/and clonal hematopoiesis and hematological malignancy (Figure 1). In fact, all types of solid and blood cancers, including MPNs, are accompanied by some degree of chronic inflammation [21, 22]. The mechanisms of inflammation in the context of cancer are complex and multiple. Chronic inflammation is an early event in many types of cancers and in certain lymphoma but in MPNs, the possibility that chronic inflammation precedes the acquisition of the main MPN mutations is a new subject of research. Whatever its chronology, chronic inflammation facilitates further DNA alteration in cancer and adjacent cells, and targeting inflammation and its causes should offer new opportunities of cancer treatment and also help reduce complications [21–23].

**Figure 2:**
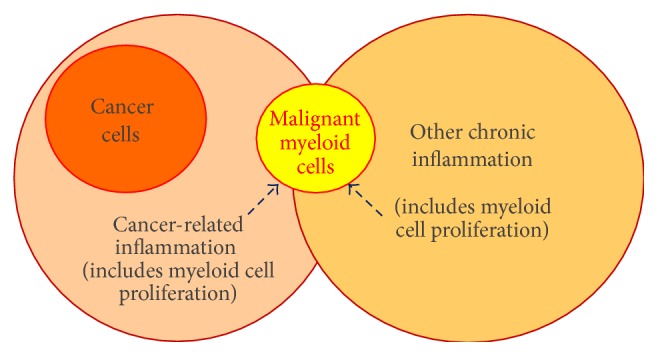
Increased risk of myeloid malignancy in case of chronic inflammation. Chronic inflammation may be related to solid cancer or to other causes (infectious, toxic, and physical). In all cases the immune response includes an increased stimulation of the production of myeloid cells, with the associated increased risk of DNA alteration in dividing progenitor cells. Over the years, a myeloid progenitor may acquire a defect in a gene critical for survival or proliferation (*MPL*,* JAK2*, and* CALR*?) and a* MPL*-,* JAK2*-, or* CALR*-mutated malignant clone may expand and lead to a MPN. Other mutations providing a mild growth advantage (*TET2*,* IDH1/2*?) may occur before or after the* MPL*,* JAK2*, or* CALR* mutations. In the case of inflammation related to solid cancer, cancer cells and the inflammatory cytokines they produce likely affect immune cells.

In the context of solid cancer, chronic inflammation may be reactive to a persistent tissue injury (exposure to toxics or to infectious agents) or/and to the tumor itself; it may also be a consequence of tumor-associated mutations or of treatment (radiotherapy or chemotherapy) (Figure 2). Thus inflammation may precede or/and accompany malignancy, and polyclonal hematopoietic cells of the myeloid and lymphoid lineages participate in the inflammation process. Whatever the cause(s) of inflammation, sustained stimulation of the proliferation of lymphoid or myeloid cells to maintain inflammation over months or years increases the risk of DNA alteration in these cells and the subsequent emergence of a mutated clone (initiation of malignancy) or of additional mutated clones (during or after radio- or chemotherapy). Figure 2 represents progression from chronic inflammation and stimulation of polyclonal myelopoiesis to clonal myelopoiesis, expansion of a mutated myeloid clone, and myeloid malignancy.

In MPNs, cells from all myeloid lineages may belong to the malignant clone: erythroid cells, megakaryocytes, neutrophils, and monocytes; B-lymphocytes or/and T-lymphocytes may be mutated too, but only rarely and usually in PMF [30]. In contrast to patients with solid cancer, for whom myelopoiesis is normal and polyclonal, the immune response in patients with MPNs includes the mobilization and activity of mutated (clonal) myeloid cells as well as of healthy myeloid and lymphoid cells. Depending on the small or large size of the MPN clone, the myeloid part of immune and inflammatory responses may be partially or mostly clonal and subsequently mildly or severely defective. This side of myeloid malignancy is often neglected but likely important in the pathogenesis and complications of MPNs.

One cause of chronic inflammation recognized as increasing the risk of malignant transformation of affected cells and tissues is chronic infection. Indeed it is now well established that latent infection can be associated with various types of solid cancer or/and with lymphoid malignancy [31–37]. In blood malignancies, two main transforming mechanisms may be at play: direct cell infection and transformation by oncogenic molecules or indirect transformation via chronic antigen stimulation and cell proliferation resulting in increased risk of acquisition of genetic defects.

### 2.1. Molecular Pathways Activated in Chronic Inflammation

**Figure 3:**
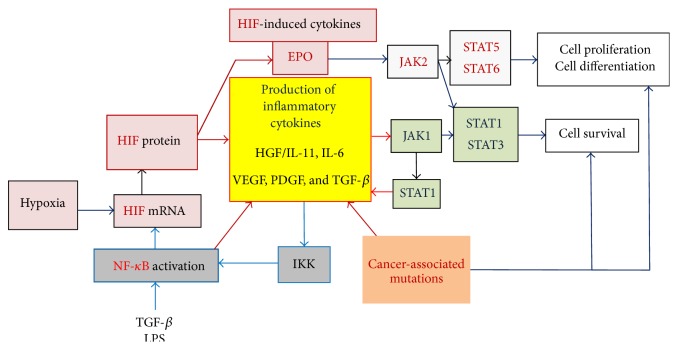
Main molecular pathways activated for the production of inflammatory cytokines. Three main transcription factors control the production of inflammatory cytokines and subsequently cell survival and proliferation: (i) HIF-1*α*, activated in hypoxic tissues, regulates the transcription of multiple genes including numerous inflammatory cytokines and growth factors that promote cell survival, fibrosis, and neoangiogenesis [12, 13]; (ii) NF-*κ*B induces the expression of many inflammation cytokines and growth factors, as well as HIF-1*α* mRNA; (iii) STAT1, like NF-*κ*B, induces the expression of several inflammation cytokines. To a lesser degree, STAT3 also regulates cytokine transcription, notably of IL-6. STAT1 and STAT3 are activated by JAK kinases, essentially JAK1, but other kinases also activate STAT transcription factors (e.g., MET, the HGF receptor). In addition, cancer-associated mutations may affect the expression (*TET2* and* IDH1/2* mutations) or signaling (*JAK2*-V617F,* CBL*, or* LNK* mutations) of cytokines or cytokine receptors. Certain growth factors (TGF-*β*) and other molecules such as liposaccharide (LPS), a component of Gram-negative bacteria, can also activate the NF-*κ*B pathway and subsequently the HIF and JAK/STAT pathways. Red arrows represent pathways that directly lead to increased production of inflammatory cytokines.

**Figure 4:**
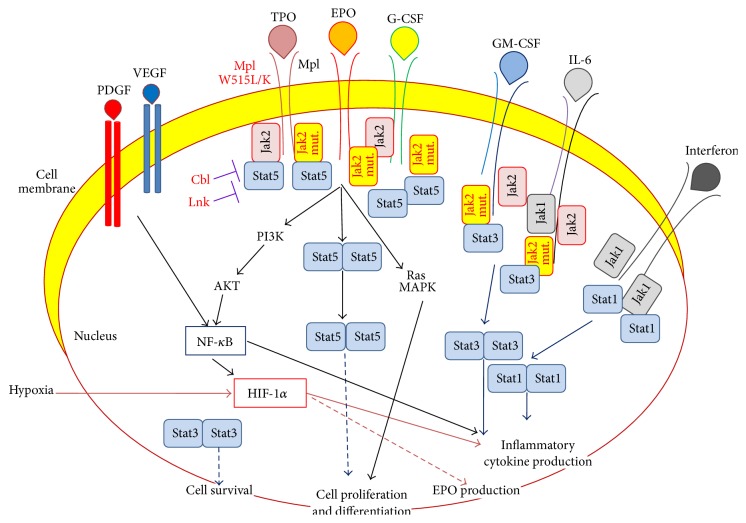
Molecular pathways activated by inflammatory cytokines and growth factors and affected by MPN-associated mutations. Most cytokine and growth factor receptors can activate the HIF-1*α*, NF-*κ*B, and/or one or more of the different JAK/STAT pathways, either directly or indirectly. Among the JAK kinases, myeloid cells express essentially JAK2 and to a lesser degree JAK1 and TYK2 (not represented). JAK2 activates STAT5 and STAT3. JAK1 activates mainly STAT1 and to a lesser degree STAT3. The different STAT transcription factors form homodimers as well as heterodimers, which allows for a differential regulation of the expression of inflammatory cytokines. In MPN clonal cells, the JAK2-coupled receptors of EPO, TPO, and G-CSF may form complexes with and activate wild type JAK2 only, V617F-mutated JAK2 only, or wild type and V617F-mutated JAK2, which likely result in different levels of activation of the JAK2/STAT pathways concerned. Moreover, EPO, TPO, and G-CSF activate other molecular pathways besides the JAK/STAT pathways, such as the antiapoptosis, prosurvival PI3K/AKT pathway and the proproliferation RAS/MAPK pathway. Of note, activation of HIF-1*α* leads to an increased production of inflammatory cytokines in all cell types, but HIF-1*α* induces EPO expression only in the rare EPO-producing cell types (renal cells, neuronal cells, and certain tumors). LNK loss-of-function mutants result in enhanced activation of JAK2/STAT5. The* CBL* mutants detected in MPNs also enhance JAK/STAT signaling. Blue arrows represent JAK/STAT pathways, and red arrows represent HIF pathways.

During inflammation, cytokines are released which signal cells such as T-lymphocytes and monocytes-macrophages to travel to the site of injury. In turn, activated immune cells increase their production of inflammatory cytokines, chemokines, hematopoietic cytokines, and other growth factors, hereby stimulating numerous cell types from their environment (fibroblasts and endothelial cells), which further increases the production of inflammatory cytokines. In this context, the nuclear factor kappa-B (NF-*κ*B) and JAK1/STAT1 pathways are the two main molecular pathways activated to enhance the production of inflammation cytokines (Figures 3 and 4) [12, 21, 38]. In case of inflammation linked to hypoxia, which may occur after thrombosis or because of cell accumulation, the production of inflammatory cytokines and growth factors by the cells exposed to hypoxia is upregulated via the HIF-1*α* pathway [39, 40]. As shown in Figure 3, the NF-*κ*B, HIF-1*α*, and JAK/STAT pathways interact closely. They act in synergy, NF-*κ*B activating the HIF-1*α* pathway, which in turn leads to increased activation of several signaling pathways, including JAK2/STAT5 (via the production of erythropoietin (EPO)), STAT3 (via inflammation cytokines from the IL-6 family or via EPO, hepatocyte growth factor (HGF), platelet-derived growth factor (PDGF), and vascular endothelial growth factor (VEGF)), and STAT1 (via type I and type II inflammatory cytokines) (Figure 4). Moreover, the level of JAK activity affects the expression of transcription factors HIF-1 and HIF-3 [13, 41]. In the context of malignancy, the genetic mutations associated with the tumor may or may not induce the production of inflammation cytokines in mutated cells. This aspect is particularly important in the context of blood cancers since the mutated cells are involved in the immune response or/and are major sources of production of inflammatory cytokines.

Situations where chronic inflammation results from more than one cause are not rare: physical injury and infection, thrombosis and hypoxia, solid cancer and infection,* JAK2*-mutated MPN and thrombosis, and so forth. The degree of activation and overall synergistic action of the three main pathways which control the production of inflammatory cytokines may vary widely, which allows for infinite qualitative and quantitative differences (Figure 4). Thus the cytokine profile and degree of overproduction of inflammatory cytokines and other mediators of inflammation are expected to vary from patient to patient, according to the cause(s) of inflammation, the cell types being stimulated, and the molecular pathways involved.

### 2.2. Main Inflammatory Cytokines, Cellular Sources, and Role in Expansion of the MPN Clone

Cytokines may be divided into four groups on the basis of their biological functions: (i) natural immunity, for TNF-*α*, IL-1, IL-6, IL-5, IL-8, and chemokines; (ii) lymphocyte activation, growth, and differentiation, for IL-2, IL-4, and transforming growth factor-*β* (TGF-*β*); (iii) regulation of inflammation, also for IL-4, TGF-*β*, and IL-1, IL-10, IFN-*γ*, and granulocyte macrophage-colony stimulating factor (GM-CSF); and (iv) stimulation of leucocyte growth and differentiation, for IL-1, IL-3, IL-5, IL-6, granulocyte-CSF (G-CSF), macrophage-CSF (M-CSF), and GM-CSF. Cytokines are also classified as Th1 (proinflammatory) cytokines (IL-1, IL-2, IL-12, TNF-*α*, and IFN-*γ*) and Th2 (anti-inflammatory) cytokines (IL-4 and IL-10, notably). Th1 cytokines cause stimulation of CD8-positive cytolytic T-lymphocytes, leading, for instance, to viral clearance. Hence the cytokines produced during chronic inflammation vary according to the cause of inflammation and the cell types involved.

The cytokines produced in large quantities during inflammation may also vary according to the molecular pathways that are being activated (due to the acquisition of mutation(s), infection, hypoxia, etc.). The cytokines produced following activation of the NF-*κ*B and JAK1/STAT1 pathways include IL-1*β*, IL-6, IL-8, IL-10, IL-11, IL-12, IL-13, IL-15, IL-22, vascular endothelial growth factor (VEGF), TNF-*α*, TGF-*β*, platelet-derived growth factor- (PDGF-) BB, b-fibroblast growth factor (FGF), G-CSF, GM-CSF, IFN-*α*, macrophage inflammatory protein- (MIP-) 1*α*, MIP-1*β*, MIP-3*α*, HGF, IFN-*γ*-inducible protein 10 (IP-10), monocyte chemotactic protein- (MCP-) 1, monokine induced by IFN-*γ* (MIG), and regulated on activation, normal T-cell expressed and secreted (RANTES) [13, 40, 41]. In case of hypoxia, increased HIF-1*α* expression leads to the upregulation of the production of EPO, VEGF, insulin growth factor 2 (IGF-2), TNF-*α*, TGF-*β*, PDGF, fibroblast growth factor (FGF) 2, IL-6, HGF, and its receptor MET (list not exclusive) [42]. Most inflammation cytokines activate the JAK1/STAT3 pathway, thus ensuring enhanced survival of many cell types, including fibroblasts, endothelial cells, and hematopoietic progenitors. Certain cytokines and growth factors activate other molecular pathways, such as the Smad proteins for TGF-*β*, JAK1/STAT1 for IFN, or the JAK2/STAT5 pathways for EPO and G-CSF, which stimulate the production of red blood cells and granulocytes, respectively [43–46].

In MPNs, several inflammatory cytokines and growth factors (IL-6, IL-8, GM-CSF, HGF, VEGF, b-FGF, and TGF-*β*) are found to be significantly overproduced in all subtypes, yet with a large variability in quantity (Table 1). Of note, TGF-*β*1 inhibits normal hematopoiesis in humans via its receptor II (TGF-*β*RII). In cancer cells, a reduced expression of TGF-*β*RII is frequent, which suggests that malignant MPN progenitors may also acquire resistance to TGF-*β*1 by downregulating TGF-*β*RII expression [47, 48]. For certain cytokines, qualitative and quantitative differences in production can be related to the MPN phenotype. Excess production of IL-4, IL-10, and TNF-*α* has been reported in ET; elevation of IL-11 levels has been described only in PV; and in PMF, many cytokines, growth factors, and chemokines are produced at high levels but IFN-*γ* levels are usually low (Table 1) [45, 49–54].

The cellular sources of production of inflammation cytokines, chemokines, and growth factors are many and of course vary depending on the MPN subtype and associated complications (thrombosis and bone marrow fibrosis). However, they usually include most of the cell types which constitute the bone marrow microenvironment or hematopoietic niche, fibroblasts, macrophages, T-lymphocytes, and endothelial cells, as well as healthy or mutated (clonal) hematopoietic progenitors and mature blood elements, platelets, neutrophils, monocytes, and macrophages [55–59]. Macrophages may present with a M1 phenotype, where they produce large amounts of TNF-*α* and IL-12 (both elevated in PV and PMF) as well as IL-23. Macrophages of the M2 phenotype secrete IL-4 or IL-10 (both elevated in ET).

In MPNs the EPO level is low and typically undetectable in PV [60]. The presence of the activating* JAK2*-V617F mutation in >95% of PV cases likely compensates for the low EPO production by rendering erythroid progenitors highly responsive to low doses of EPO, to result in polycythemia. This explanation is likely also valid for the 50–60% of ET and PMF which are* JAK2*-V617F-mutated. Intriguingly, blood levels of other cytokines which also activate the JAK2/STAT5 pathways (TPO and G-CSF) or the JAK2/STAT3 pathways (GM-CSF, IL-12, IL-33, and cytokines of the IL-6 family: IL-6, IL-11, and oncostatin M (OSM)) are often normal or elevated in MPNs (Table 2). Several of these cytokines are produced by nonhematopoietic cells and also by myeloid progenitors, and they promote the survival and proliferation of both clonal and nonclonal myeloid progenitor cells. This is the case notably for TPO, IL-6, IL-8, IL-11, IL-33, GM-CSF, HGF, and TNF-*α* and several of these cytokines have been proven to contribute to the expansion of the* JAK2*-V617F-mutated cells [51–53, 59, 61]. Regarding TPO, it is important to note that the low surface expression of Mpl (the TPO receptor) observed in MPN progenitors and platelets likely limits the effect of high circulating levels of TPO. The reasons for the low expression of Mpl in MPN patients are not fully understood. On one hand,* JAK2*-V617F is thought to be less efficient than wild type JAK2 to bring Mpl receptors to the cell surface and possibly to increase Mpl destruction. On the other hand, a high TPO level and activating* JAK2*-V617F or* MPL*-W515L/K mutations may be ways of counteracting Mpl repression in progenitor cells.

Another intriguing observation is the elevated production of IL-33. IL-33 is an alarmin known to help fight viral infection that is implicated in autoimmunity, and an increased risk of autoimmune disease has been reported in MPN patients [61, 62]. Chronic stimulation by the above cytokines also facilitates the survival and expansion of fibroblasts and fibrosis (IL-6 and b-FGF), monocytes-macrophages (IL-6 and GM-GSF), and platelet production (IL-6) and neoangiogenesis (VEGF), whereas IL-12 and IL-33 activate T-lymphocytes and natural killer (NK) cells. In addition, MPN cells accumulate in the bone marrow, which leads to some degree of hypoxia and subsequent activation of the HIF-1*α* pathway, with upregulation of STAT3 expression and production of cytokines which further promote cell survival (IGF-2, HGF, and IL-6), fibrosis (PDGF, FGF2, and IL-6), and neoangiogenesis (VEGF) [42, 63].

Altogether, the qualitative and quantitative differences found in cytokine production in the three MPNs subtypes hint that the causes and mechanisms of chronic inflammation likely differ in ET, PV, and PMF. The* JAK2*-V617F,* MPL*-W515L/K, and* CALR* mutations likely influence clinical symptoms but do not explain differences in inflammation. For instance,* JAK2*-V617F,* MPL*, and* CALR* mutations are detected at similar levels of expression in ET (associated with mild or very mild inflammation) and in PMF (characterized by severe inflammation). Thus it is important to investigate and understand the mechanisms of inflammation at play in each MPN subtype, including those independent of* JAK2*,* MPL*, or* CALR* mutations.

### 2.3. Main Clinical and Biological Symptoms

The main clinical symptoms observed in MPNs which are linked to an increased production of inflammation cytokines are fatigue, fever, itching, night sweats, weight loss, and, to some extent, splenomegaly. These symptoms are frequent in PMF; they occur in PV but are mostly absent in ET, which is a good reflection of the degree of production of inflammation cytokines characteristic of PMF (high or very high), PV (moderate to high), and ET (mild).

The main biological parameters routinely assessed which are affected in case of inflammation include blood cell counts (in particular leukocyte, neutrophil, and platelet counts), iron levels, and several proteins: the C-reactive protein (CRP), haptoglobin, alpha-1 acid glycoprotein (orosomucoid), ferritin and fibrinogen (increased), and albumin and transferrin (decreased). The major stimulus of increased synthesis by liver (hepatocytes) is IL-6. These inflammatory proteins present with different kinetics: inflammatory positive markers which are increased early include CRP, haptoglobin, and alpha-1 acid glycoprotein, whereas fibrinogen, ferritin, and transferrin are late-acting inflammatory proteins.

Elevation of the leukocyte and platelet counts is typical of MPNs and thus does not allow distinguishing between inflammation and MPN. CRP is elevated in MPNs, particularly in PMF, and pentraxin 3 has been reported to decrease in MPNs [64]. A high CRP and low pentraxin 3 were linked to a high risk of thrombotic events in PV and in ET, and a high CRP was associated with shortened leukemia-free survival in MPN patients with myelofibrosis [64, 65]. The level of IL-6 in serum is almost constantly increased in case of MPN but IL-6 levels (and other inflammatory cytokines) are not measured in routine laboratory practice.

## 3. Activation of the Molecular Pathways of Inflammation by MPN-Associated Mutations

MPNs are characterized by the activating* JAK2*-V617F and* MPL*-W515L/K mutations, the* CALR* mutations, and also high levels of total Jak2 (wild type and V617F-mutated) in neutrophils and platelets [3–10]. The effect of* JAK2*-V617F mutation is to activate primarily the STAT5 pathways but the STAT3 pathways are also activated (Figure 4) [66]. The* MPL*-W515L/K mutations presumably stabilize Mpl, the Jak2-coupled dimeric receptor for TPO [67]. TPO is known to stimulate the JAK/STAT pathways and also PI3K/AKT, ERK, p38, NF-*κ*B, and HIF [67–69]. Accordingly, in transfected cells expression of* MPL*-W515L/K mutants resulted in increased activation of ERK (extracellular signal-regulated kinases) 1 and ERK 2 (ERK1/2) and AKT (protein kinase B) in absence and in presence of TPO [5, unpublished observations]. To our knowledge, the effect of* MPL*-W515L/K mutations on NF-*κ*B and HIF has not been studied. In any case, the* JAK2*-V617F mutation activates STAT3 and the* MPL*-W515L/K mutations activate STAT1 and STAT3, which implies that they may stimulate the production of inflammatory cytokines (Figure 4). However, in MPN progenitor cells and platelets, the expression of Mpl receptors at the membrane surface is often very low, which likely attenuates the effect of TPO stimulation and* MPL*-W515L/K mutation.

The molecular pathways possibly activated by* CALR* mutations remain unclear. Calreticulin is a calcium-binding protein chaperone normally located in the endoplasmic reticulum (ER); the* CALR* mutations associated with MPNs all result in C-terminal truncated forms of calreticulin located in the cytosol. Thus it is presumed but not formally demonstrated that* CALR* mutants may affect intracellular calcium flux as well as the trafficking and secretion of glycoproteins, which could potentially lead to altered expression and activation of cytokines, receptors, Jak2, and other signaling molecules. Consistently, the initial papers reported an activation of the JAK2/STAT5 pathway in transfected cells which expressed* CALR* exon 9 mutants [8, 9]. However, the precise molecular mechanisms linking* CALR* mutants and the JAK2/STAT5 pathway have not been identified.

More rarely, in ET or PMF the “driving” mutation may be a loss-of-function mutation in the* LNK* gene or in the* CBL* gene [70–74]. LNK is an adaptor protein which acts as a negative regulator of TPO/Mpl-mediated activation of JAK2. Expression of LNK loss-of-function mutants also results in enhanced activation of the JAK2/STAT5 pathway.* CBL* codes for an E3-ubiquitin ligase which promotes the ubiquitination of signaling molecules, including tyrosine kinases. The* CBL* mutations detected in MPNs cause the loss of E3-ubiquitin ligase activity, thus resulting in increased signaling and cell proliferation. So far there is no evidence that* LNK* or* CBL* mutations induce the production of inflammatory cytokines, but they may alter their signaling. Figure 4 summarizes the pathways activated by the main MPN-driving mutations.

Mutations in the* TET2*,* IDH1*,* IDH2*,* EZH2*,* ASLX1*, or* DNMT3A* genes may also be found in MPNs. They are not specific of MPNs: they are found also in other blood and solid malignancies. Their main action is to alter the regulation of gene expression [75–83].* TET2* and* IDH1/2* mutants impair the hydroxylation of methylcytosine and thus affect DNA methylation. More precisely,* TET* gene products catalyze the conversion of 5-methylcytosine to 5-hydroxy-methylcytosine (5-OH-MeC), a reaction that depends in part on iron and oxygen [80, 81].* EZH2* (enhancer of zeste homolog 2) gene codes for a histone methyl transferase, and* ASXL1* (additional sex combs like transcriptional regulator 1) gene product belongs to the Polycomb group of proteins and thus is thought to disrupt chromatin and alter gene transcription.* DNMT3A* codes for a DNA methyltransferase and mutations presumably alter the epigenetic regulation of gene expression [82]. Thus one cannot exclude that these mutations may alter the expression of genes coding for inflammatory cytokines or receptors. Interestingly, some of these mutations have been shown to precede* JAK2*-V617F [15].

## 4. Inflammatory Cytokines Produced as a Consequence of MPN-Associated Mutations

Not surprisingly,* JAK2*-V617F has received most of the attention. Several groups have studied the production of inflammation cytokines in* JAK2*-V617F-mutated cells or in murine* JAK2*-V617F-driven MPN models. So far published reports concluded that,* in vitro*,* JAK2*-V617F can increase the production of IL-6, IL-8, IL-9, OSM, CCL3, CCL4, and TNF-*α* [53, 59, 84, 85]. However, in MPN patients there is no correlation between the* JAK2*-V617F burden and the blood or serum levels of these cytokines. In fact, it is highly probable that only a fraction of these cytokines is under the control of* JAK2*-V617F. Firstly, IL-6, IL-8, and OSM are abundantly produced by nonhematopoietic (nonclonal and nonmutated) cells [51–53]. Secondly, certain molecules produced under the control of* JAK2*-V617F, such as OSM, in turn stimulate the production of other inflammatory cytokines in a* JAK2*-V617F-independent manner [85]. Thirdly, in the* JAK2*-V617F^+/+^ HEL cell line, anti-JAK2 miRNA experiments had only a partial inhibiting effect on IL-6 mRNA expression; in these experiments, anti-JAK2 miRNA experiments had no effect on the expression of IL-11 and HGF [53]. Thus in* JAK2*-V617F-mutated cells, major inflammatory cytokines may be controlled partially (IL-6) or totally (IL-11 and HGF) by molecular pathways not regulated by* JAK2*-V617F.

Regarding* MPL*-W515 mutations, only one group reported the analysis of inflammation cytokines produced in* MPL*-W515L-mutated cells, in a murine bone marrow transplantation model: expression of* MPL*-W515L was associated with a significant increase in the production of IL-6, IL-10, IL-12 (p40), TNF-*α*, CSF3, and chemokines CCL2, CXCL9, and CXCL10 [84]. Again,* MPL*-W515L-mutated cells were not the sole source of production of these cytokines.

Regarding the* CALR* exon 9 mutations associated with MPNs, their effect on cytokine expression is not known. It is interesting to note that soluble calreticulin has been reported associated with increased production of IL-6 and TNF-*α* [86].

Regarding* TET2*,* IDH1/2*, and* ASXL1* mutations, it was reported that mutated forms of IDH1/2 were associated with specific DNA hypermethylation profiles, and the list of genes found to be differentially methylated includes several genes linked to inflammation, particularly the IL11-R*α* and TGF-*β*RI receptors [79]. Interestingly, IL-11 and TGF-*β* are secreted at high levels in case of inflammation and both alter myelopoiesis. IL-11R*α* is also differentially methylated in* TET2*-mutated cells [79]. Hypermethylation of the genes encoding IL11-R*α* and TGF-*β*RI receptors would presumably lower their expression and subsequently make clonal progenitor cells less sensitive to the inhibiting action of TGF-*β* and anti-inflammatory action of IL-11. Since* TET2* and* IDH1/2* mutations are mostly found in PMF, it is possible that these mutants play a role in the aggravation of inflammation observed in severe forms of PMF [87, 88]. In myelodysplastic syndromes,* ASXL1* mutations combined with* SETBP1* mutations were reported to repress the TGF-*β* pathways [89]. However no study of cytokine or receptor protein expression in relation to* ASXL1* mutation in MPNs has been published.

## 5. Inflammatory Cytokines Produced as a Consequence of Germline Genetic Defects

Germline defects, variants, or haplotypes can affect, directly or indirectly, the expression or signaling of inflammatory cytokines and receptors, thus potentially attenuating or aggravating chronic inflammation. The 46/1 (*JAK2* GGCC) haplotype and single-nucleotide polymorphisms (SNP) in* JAK2*, in the telomerase reverse transcriptase (*TERT)*, in the MDS1 and EVI1 complex locus (*MECOM*), or in* HBS1L-MYB* have been reported to be associated with a predisposition to mutation in the* JAK2* gene on the same allele (*JAK2* GGCC haplotype) or a predisposition to the development of a MPN (*MECOM*,* TERT*,* JAK2*, and* HBS1L-MYB* variants) [90–94]. To this day it remains unclear how these hereditary genetic variants act to facilitate the development of MPNs, but alteration of the transcription of the concerned genes is possible. Regarding germline* JAK2* variation, inappropriate expression of* JAK2* would clearly disturb myelopoiesis and alter the contribution of myeloid cells to inflammation responses. Consistently, the* JAK2* GGCC haplotype was reported to be associated with a defective response to cytokine stimulation, increased risk of inflammation, and impaired defense against infection [95, 96]. In CML, cells with short telomere length were found to express a specific “telomere-associated” cytokine and chemokine secretory phenotype [97]. Little is known on the functional effects of MECOM variants on cytokine production but Yasui et al. recently reported that the EVI1 oncoprotein could alter TGF-*β* signaling and TGF-*β*-mediated growth inhibition [98, 99].

It is established that variations due to SNPs in the promoter region of genes coding for inflammatory cytokines and receptors potentially affect their production. Many groups have published SNPs associated with an altered production of a cytokine or a cytokine receptor, and such SNPs concern all the main cytokines involved in inflammation: IL-1, IL-1R*α*, IL-2R, IL-6, IL-8, IL-10, IL-12, IL-33, TNF-*α*, HGF, and MCP1/CCL2 [100–115]. SNPs have been shown to control the expression of these cytokines* in vitro* and individuals who carry the SNP are described as high or low producers [116–118]. Cytokine polymorphisms have been studied in association with specific diseases, response to infectious agents, or immune response to inflammation. To our knowledge, this type of analyses has never been performed in MPNs.

## 6. Clonal and Nonclonal Chronic Inflammation in MPNs

Chronic inflammation associated with MPN may have several causes, and their recognition should allow offering improved and individualized treatment to MPN patients.

### 6.1. MPN-Related Chronic Inflammation

#### 6.1.1. Clonal Inflammation

As described above, part of the inflammation is clonal since MPN clonal cells produce inflammatory cytokines (IL-6, IL-8, IL-9, IL-11, OSM, TNF-*α*, CCL3 (MIP-1*α*), and CCL4 (MIP-1*β*)); the eventual acquisition of* IDH1/2* or* TET2* mutations may aggravate “clonal inflammation” by altering the expression of certain receptors (IL-11R*α*, TGF-*β*R1). The consequences are multiple: (i) enhanced survival and growth of clonal cells (IL-6, IL-8, IL-11, and TNF-*α*); (ii) increased production of inflammation cytokines that target bone marrow stromal cells as well as hematopoietic progenitors, via the action of OSM, IL-11, and IL-6; (iii) resistance of clonal cells to growth inhibitors, via a reduced expression of IL-11R*α* or TGF-*β*R1 [53, 59, 61, 63, 119, 120]. In addition, clonal cells can recruit and activate neutrophils, monocytes, and natural killer cells via the production of CCL3 and CCL4; the neutrophils and monocytes potentially recruited may be clonal or non-clonal.

#### 6.1.2. Nonclonal, Reactive Inflammation

Any malignant process induces nonclonal immune responses which aim to restrict and eventually destroy the malignant cells. In case of advanced disease, clonal cells accumulate and nonclonal, hypoxia-induced inflammation can develop. Nonclonal inflammation may also be reactive to treatment. In MPNs, the mature myeloid cells which participate in the antitumoral or hypoxia-induced or therapy-related “reactive” inflammation response may be clonal or nonclonal. Depending on the MPN subtype, the size of the clonal population is likely to be large (PV and PMF) or moderate or small (ET), implying that the clonal part of reactive inflammation is probably more significant in PMF and PV than in ET. This should not be overlooked because clonal cells likely mount a less efficient immune response than healthy cells, meaning that the inflammation/immune response could be rather inefficient in PV and PMF. Consistently, an increased risk of a second cancer was reported in MPN patients [121].

### 6.2. Chronic Inflammation and Myeloid Stimulation as Predisposition to MPNs

The observation that major inflammatory cytokines are produced independently of MPN-associated mutations and the demonstration that* JAK2*-V617F can be a late event in MPN development are consistent with the hypothesis that chronic stimulation of myelopoiesis (via inflammation) may precede the acquisition of mutation in the* JAK2* (*MPL* and* CALR*?) gene(s) in subsets of MPN patients. A frequent objection is the lack of evidence of inflammation or myeloid stimulation prior to the diagnosis of a MPN. However, it is not rare that routine blood tests of patients, especially older patients, reveal a slight elevation of leukocyte or platelet counts, or hematocrit, with or without biological evidence of mild inflammation. There are dozens of reasons for mild alterations of blood counts, ranging from smoking, stress, obesity, and diverse latent infections to mild forms of chronic inflammatory diseases (intestinal, rheumatoid, skin, type 2 diabetes, atherosclerosis, etc.). Such patients are simply observed; investigation begins when blood counts rise significantly (reach at least one of the WHO criteria of MPN) or when patients present clinical symptoms related to MPN or to the underlying cause of chronic myeloid stimulation or inflammation. Also, it is not rare to detect lymphoid infiltrates in the bone marrow of MPN patients and monocytosis or lymphopenia in peripheral blood, sometimes prior to the diagnosis of MPN; these observations may be considered as evidence of a disturbed immune response. Thorough investigation of the stages preceding the diagnosis of overt MPN, similar, for instance, to the studies that established monoclonal gammopathy of undetermined significance (MGUS) as the precancerous stage of multiple myeloma, is needed in MPNs to validate the hypothesis of chronic (antigen-mediated or not) stimulation of myelopoiesis preceding the acquisition of* JAK2*,* MPL*, or* CALR* mutation.

The chronic inflammation and myeloid stimulation hypothesis is attractive, because it can explain several if not all of the mysteries that persist in MPNs. For instance, chronic myeloid stimulation allows the recurrent acquisition of* JAK2*-V617F, multiple* JAK2* mutations, and combinations of* JAK2*,* MPL*, or* CALR* mutations regularly reported in MPNs. Early chronic inflammation and myeloid stimulation would explain that* JAK2*-V617F burden and clinical symptoms and disease severity are not correlated. The recent reports that patients under treatment with JAK inhibitors may develop or reactivate viral infection, possibly due to impaired NK cell function, are also consistent with chronic infection contributing to the inflammation associated with MPNs [122]. Importantly, the chronic stimulation hypothesis allows for multiple causes of inflammation, infectious or not, some mild (as observed in ET) and some severe (as typical of PMF). Last but not least, the chronic myeloid stimulation hypothesis allows for many different initial causes and thus would explain why the* JAK2*-V617F mutation and to a lesser degree the* MPL* exon 10 and* CALR* exon 9 mutations are associated with very different diseases (ET, PV, PMF, RARS-T, and splanchnic thrombosis for* JAK2*-V617F; ET, PMF, and RARS-T for* MPL* and* CALR* mutations). For all these reasons, chronic myeloid stimulation and inflammation, and notably latent infection, deserve investigation as initial, early, or complicating events of MPNs.

Indeed chronic exposure to various toxic compounds or to infectious pathogens and subsequent chronic inflammation frequently precedes malignant cell transformation in the context of solid cancers; importantly, the same toxics or infectious agents associated with solid cancer may also lead to lymphoid malignancy (Table 2) [31–37]. Normal immune responses following infection include the stimulation of myelopoiesis (granulocytes and monocytes). The production of B-lymphocytes and plasma cells is stimulated to produce polyclonal Ig. If infection becomes chronic, a focusing of the Ig response from polyclonal to monoclonal (mc) immunoglobulins (Ig) may occur, and that will persist as long as the infection. Epstein-Barr virus (EBV), Hepatitis C virus (HCV), Hepatitis B virus (HBV), or* Helicobacter pylori* (*H. pylori*) stimulate polyclonal B-cell proliferation, and these pathogens are implicated in several B-cell malignancies (Burkitt, Hodgkin, non-Hodgkin lymphoma, and chronic lymphocytic leukemia) via cell infection and direct transformation (EBV and HCV), via antigen-driven stimulation (*H. pylori*), or both (EBV and HCV) [32–37]. Moreover, EBV, cytomegalovirus (CMV), HHV-8, and HHV-6 can induce a chronic monoclonal Ig response [123–126].

In support of the hypothesis that infection may predispose to chronic hematological malignancy, we showed that, for about 25% of patients with multiple myeloma, the purified mc Ig specifically recognizes an antigen from HCV, EBV, or* H. pylori* [124–127]. These important findings suggest that infectious agents may also initiate multiple myeloma, not just certain types of lymphoma, which opens new possibilities of curative treatment, as demonstrated recently by the regression of one case of HCV-associated myeloma following treatment by IFN-*γ*  [128]. Antigen-driven proliferation as a facilitator of DNA mutation acquisition and cell transformation is rarely investigated in the context of myeloid malignancies but since chronic antigen stimulation also concerns myeloid cells, latent infection as a cause of inflammation in chronic myeloid disorders should not be excluded. Thus a promising research approach for chronic myeloid disorders is to propose that, for subsets of patients, malignancy may result from chronic, polyclonal abnormal immune response by myeloid cells, eventually facilitating excessive myeloid proliferation, acquisition of genetic alterations in genes that are critical for myelopoiesis (*JAK2* and* MPL*;* CALR*?), and transformation of progenitor cells from the most stimulated lineage(s) and then expansion of a malignant clone.

## 7. Consequences for the Treatment of MPNs

Logically, the* JAK2*-V617F mutation rapidly became the main target of treatment in MPNs after its discovery in 2005. In contrast, chronic inflammation has so far been neglected in the treatment of these diseases.

Recognition of the importance of inflammation in the pathogenesis of MPNs offers great opportunities to improve therapy. The JAK inhibitor trials showed that blocking JAK2 function significantly reduced inflammatory cytokine levels and other markers of inflammation in PMF patients, resulting in improved clinical symptoms. Patients benefited from JAK inhibitors even when the* JAK2*-V617F mutant burden was not reduced or when their disease was not associated with* JAK2* mutation. Although the comprehension of the causes and mechanisms of inflammation in MPNs is still very incomplete, accumulated knowledge indicates that NF-*κ*B and JAK1 are major pathways for the production or/and signaling of inflammatory cytokines. In certain cases, the HIF-1*α* pathways may also be activated. The three pathways are closely linked (Figures 3 and 4), and used alone, inhibitors of the JAK/STAT pathways (or inhibitors of NF-*κ*B) cannot be expected to completely block cytokine production and signalling in MPNs. In a murine model of* JAK2*-V617F-mutated MPN, selective STAT blocking resulted in increased inflammation and thrombocytosis [129]. In fact, alteration of STAT3 function (deletion or hyperactivation) is known to lead to altered myelopoiesis and increased expression of STAT1 and inflammatory cytokines, notably IL-6, a strong stimulant of platelet production, fibroblast proliferation, and inflammatory acute phase protein production [130–132]. In support of this mechanism, Grisouard et al. reported increased expression of STAT1 and STAT1 target genes in* JAK2*-V617F mice after STAT3 deletion; IL-6 and other inflammatory cytokines were not measured in this study [129].

**Figure 5:**
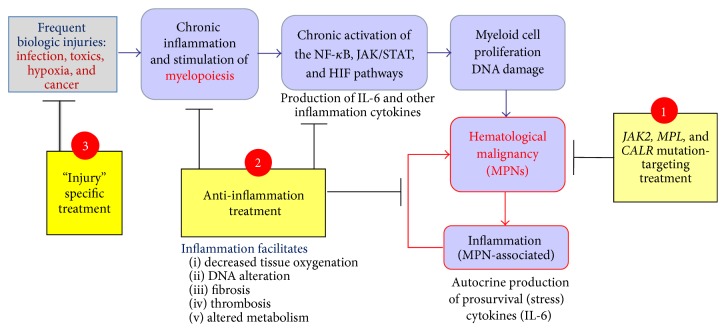
Chronic inflammation in myeloid neoplasms and new therapeutic options. In MPN patients, chronic inflammation includes the participation of* JAK2*-V617F-,* MPL*-W515 L/K-, or* CALR*-mutated cells and the production of inflammation cytokines under the control of these mutations. Chronic inflammation may also be reactive to the MPN clone or to other coexisting causes of inflammation (hypoxia due to cell accumulation in the bone marrow; thrombosis; infection; others). Healthy and mutated (clonal) myeloid cells participate in MPN-associated reactive inflammation, and the NF-*κ*B, JAK/STAT, and HIF pathways are chronically activated in the MPN clone and in cells from the bone marrow environment. Ideally treatment should combine the following: (1) inhibition of the* JAK2*-V617F,* MPL*-W515L/K, or* CALR* mutations, possible with JAK inhibitors; (2) inhibition of chronic inflammation, via the neutralization or inhibition of inflammation cytokines or receptors, and/or the inhibition of the NF-*κ*B and HIF pathways; (3) in cases where chronic inflammation precedes mutation, and a cause is identified, adequate treatment of the initial cause of inflammation could be added (e.g., antibiotics or antiviral treatment in case of latent infection).

Ideally to cure a MPN, one should aim to reduce the effects of the* JAK2*,* MPL*, or* CALR* mutant carried by the MPN clone, as well as the production and signaling of the main inflammation cytokines produced by the patient. This can be achieved by blocking the three main pathways responsible for cytokine production (these include the JAK1/JAK2 pathways) and by suppressing the cause(s) of MPN mutation, when identified. Used alone, JAK1/2 inhibitors have the capacity to block the* JAK2*-V617F and* MPL*-W515L/K mutations and a large fraction of the production and signaling of inflammatory cytokines. But for complete treatment of inflammation and mutations in MPNs, the addition of NF-*κ*B and HIF-1*α* inhibitor drugs should benefit patients [133–138]. This contrasts with myeloma, a disease not driven by the activation of the JAK2/STAT pathways where NF-*κ*B and HIF-1*α* inhibitors used alone can reduce both disease and inflammatory cytokines [139]. Another advantage of such combination therapies would allow lowering the dose of each drug and hopefully reduce toxicity. Of note, one reason why IFN-*α* is able to induce a complete clinical, biological, and molecular remission (*JAK2*-V617F-negativation) in PV and in ET patients is that IFN-*α* acts on several JAK/STAT pathways as well as on other pathways critical for the production of inflammatory cytokines [140, 141]. In short, as represented in Figure 5, the ideal MPN therapy may combine the following: (1) inhibition of the JAK1 pathway and* JAK2*-V617F,* MPL*-W515L/K, or* CALR* mutations with a JAK1/2 inhibitor and (2) NF-*κ*B and HIF inhibitors (note that (1) and (2) may be achieved with IFN-*α*). Whenever an early cause of chronic inflammation is identified, adequate treatment should be added: for instance, antibiotics or antiviral treatment in case of latent infection.

The complexity of inflammation in MPNs should not discourage attempts to define it biologically at the time of diagnosis, prior to therapeutic decisions, and during treatment monitoring. Knowing the precise inflammation status of each MPN patient would greatly help improve his/her treatment. As described earlier, the inflammation status and cytokine profile of a MPN patient are expected to vary according to the MPN subtype, presence of* JAK2*,* MPL*, or* CALR* mutation, eventual cause of inflammation preceding MPN-driving mutation, and personal genetic background. Yet what matters for therapy is the resulting cytokine profile of the patient, and nowadays establishing the inflammation cytokine profile of an individual is technically simple and not overly expansive and requires only a blood sample. Knowing that a patient is a strong producer of IL-6, HGF, or TNF-*α*, for instance, would allow focusing treatment on the target cytokine(s), perhaps by adding to the patient's combination therapy one of the existing antagonist drugs or neutralizing antibodies that specifically block these cytokines or receptors [119, 142–144].

Last but not least, extensive genetic studies and murine models have not succeeded to fully explain most of the chronic hematological malignancies, including MPNs. This suggests that genetic aberrations, although crucial, are probably not sufficient for a lymphoid or myeloid malignancy to develop, and more attention is now given to the hematopoietic niche and cytokine production, the human microbiome and oncogenic infectious pathogens, and the host's immune response [145, 146]. There is no reason to limit these important pathogenic mechanisms to lymphoid malignancies and solid cancer, and perhaps the next major research effort in the MPN field should be to investigate the validity of the hypothesis of chronic inflammation/myeloid stimulation preceding mutation acquisition. More specifically, a systematic search for latent infection in MPN patients is feasible and simple, thanks to various tests based on the multiplexed antigen or peptide microarray technology; these assays require only a small blood sample [127, 147]. Obviously the identification of an infectious cause of inflammation in subsets of MPN patients would offer additional possibilities of combined treatment (with antibiotics or antiviral therapy) (Figure 5). Regarding research and animal models of MPNs, it is possible to develop new murine models of chronic myeloid stimulation, antigen-mediated or not [148].

In conclusion, inflammation is very complex yet there are relatively simple laboratory tools to diagnose and characterize inflammation in patients. Several predictive inflammation markers are already identified in MPNs, and potent drugs that target the molecular pathways of inflammation or the inflammatory cytokines detected in excess in patients already exist. Designing new, more complete, and individualized combination treatments that include drugs that block MPN mutations as well as the main inflammation pathways is possible, and such protocols should benefit MPN patients.

## Figures and Tables

**Table 1:** Qualitative differences in inflammatory cytokine expression in ET, PV, and PMF.

MPN subtype	Cytokines produced in excess	Main cellular sources
All MPNs	IL-6, IL-8, IL-2, soluble IL-2R, HGF, TNF-*α*, TGF-*β*, GM-CSF, VEGF, and bFGF	Bone marrow fibroblasts, endothelial cells, monocytes, macrophages, T-lymphocytes, hematopoietic progenitors, and hepatocytes

ET	IL-4, IL-10, IFN-*γ*, MCP-1, PDGF-BB, and soluble IL-6R (gp80)	M2 macrophages, platelets, and T-lymphocytes

PV	IL-11, IL-12, IL-13, IL-5, and IL-7	Bone marrow fibroblasts, T-lymphocytes, M1 macrophages, and hematopoietic progenitors

PMF	IL-1*β*, IL-10, IL-12, IL-13, IL-15, IL-33, G-CSF, IFN-*α*, MIP-1*α*, MIP-1*β*, IP-10, MIG, MCP-1, and PDGF-BB	Bone marrow fibroblasts, activated T-lymphocytes, neutrophils, macrophages, hematopoietic progenitors, megakaryocytes, and platelets

**Table 2:** Infectious pathogens and toxic compounds known to be associated with chronic inflammation and solid and blood cancers.

Exposure to infectious pathogens	Affected cells	Associated solid cancer	Associated hematological malignancy
Human T-cell leukemia virus 1 (HTLV-1)	T-lymphocytes		T-cell leukemia T-cell lymphoma
Hepatitis C virus (*Flavivirus*)	Hepatocytes B-lymphocytes	Hepatocarcinoma	B-cell lymphoma, plasma cell leukemia, MGUS, and myeloma
Hepatitis B virus (Hepadnavirus)	Hepatocytes	Hepatocarcinoma	
Epstein-Barr virus (human herpes virus 4)	B-lymphocytes	Nasopharynx	B-cell lymphoma MGUS, and myeloma
Human herpes virus 8 (herpes virus)	Fibroblasts B-lymphocytes	Sarcoma	B-cell lymphoma, myeloma, and plasma cell leukemia
Human herpes virus 2 (herpes virus)	Epithelial cells	Genital cancer	
Human papilloma (papilloma virus)	Epithelial cells	Genital cancer	
*Helicobacter pylori *(bacterium)	Epithelial cells	Gastric carcinoma	MALT lymphoma, MGUS, and myeloma
*Borrelia burgdorferi *(bacterium)	Keratinocytes, fibroblasts, dendritic cells, T-lymphocytes, and monocytes/macrophages		Skin lymphoma
*Campylobacter jejuni *(bacterium)	Intestinal cells		Immunoproliferative small intestine disease (IPSID)
*Chlamydia psittaci *(bacterium)	Nasal and pulmonary cells		Ocular adnexal lymphoma
*Toxoplasma gondii (protozoon)*	Macrophages		Intraocular B-cell lymphoma
*Schistosoma haematobium *(Platyhelminthe, Trematoda)		Bladder carcinoma	
*Opisthorchis viverrini *(Platyhelminthe, Trematoda)		Bile duct carcinoma	
*Porphyromonas gingivalis* (bacterium)		Gingival/mouth cancer, pancreatic cancer?	

Exposure to toxics	Affected cells	Associated solid cancer	Associated hematological disorder or malignancy

Tobacco	Epithelial cells	Lung cancer Bladder cancer Others	Chronic stimulation of myelopoiesis
Asbestos fibers (results in asbestosis, an inflammatory condition)	Mesothelial cells	Mesothelioma	
Pesticides	All		Lymphoma
Benzene	All	Bladder cancer	Chronic myelogenous leukemia and other myeloid and lymphoid malignancies
